# Application of Blue Honeysuckle Powder Obtained by an Innovative Method of Low-Temperature Drying in Skincare Face Masks

**DOI:** 10.3390/molecules26237184

**Published:** 2021-11-26

**Authors:** Emilia Klimaszewska, Malgorzata Zieba, Klaudia Gregorczyk, Leszek Markuszewski

**Affiliations:** 1 Department of Cosmetology, Faculty of Medical Sciences and Health Sciences, Kazimierz Pulaski University of Technology and Humanities in Radom, Chrobrego 27, 26-600 Radom, Poland; e.klimaszewska@uthrad.pl (E.K.); k.gregorczyk@uthrad.pl (K.G.); 2 Department of Industrial Chemistry, Faculty of Chemical Engineering and Commodity Science, Kazimierz Pulaski University of Technology and Humanities in Radom, Chrobrego 27, 26-600 Radom, Poland; 3 Department of Medicine, Faculty of Medical Sciences and Health Science, Kazimierz Pulaski University of Technology and Humanities in Radom, Chrobrego 27, 26-600 Radom, Poland; l.markuszewski@uthrad.pl

**Keywords:** cosmetics, masks, fruit powders, blue honeysuckle, *Lonicera caerulea* fruit

## Abstract

Traditional technologies applied for obtaining plant raw materials for cosmetic production are based primarily on high-level processing, which is reflected in the qualitative composition of the resulting materials. By using low-temperature drying, it is possible to retain in the raw materials a range of valuable ingredients. In this study, blue honeysuckle powder was used as an ingredient of cosmetic face masks. The stability of the masks was evaluated. Dynamic viscosity, yield point and texture analysis of the cosmetics was performed. The color of the emulsions and the level of skin hydration after face mask application was determined. Emulsions were found to be stable. A decrease in dynamic viscosity of the emulsions as a function of increasing concentrations of the additive and under the conditions of rising rotational speed were observed. Similarly, an increase in the concentration of blue honeysuckle in the emulsions resulted in a decrease in the value of the yield point. Based on the results, it can be stated that the addition of blue honeysuckle caused a decrease in hardness of the masks, while the opposite trend was observed for adhesive force. It was found that an increase in the concentration of blue honeysuckle gave a reddish-yellow color to the samples. Corneometric assessment confirmed proper skin hydration after the application of the emulsions.

## 1. Introduction

The cosmetics industry is constantly evolving. Scientists, dermatologists as well as cosmetic companies are actively working on new cosmetic products to achieve the best possible results. There is a broad range of cosmetics with natural raw materials available on the market, and products containing ingredients of plant origin have been enjoying considerable popularity. Skincare masks are a type of cosmetic that has been attracting a great deal of attention, mainly because of a high content of active ingredients. According to a literature review [1,2,3,4,5,6,7,8,9,10,11,12,13] there is no strict classification of cosmetic masks. However, three division criteria are most commonly adopted: the physical form, the effects on the skin, and the type of skin for which masks are intended as a skin care product. Taking into consideration the form, the following mask types can be distinguished: solid, soft gel, foam, fibrous, thermo-modeling, soft powder, paraffin, and dual—with a consistency of gel and cream (manufactured in separate packaging and combined during application). Based on their effects on the skin, cosmetic masks can be divided into moisturizing, soothing, astringent, lifting-modeling (thermo-modeling), healing, under-eye (algae, gel, gel patches), warming, regenerating, and cleansing types [1,2,3,4,5,6,7,8,9,10,11,12,13,14]. Another criterion for classifying cosmetic masks is the type of skin for which they are designed. For example, skincare masks for dry, oily, and combination skin, as well as rosacea, mature and normal skin types are available. A fourth division of cosmetic masks was also proposed, which is based on how they are applied. Accordingly, the researcher identified masks which are applied cold, hot, and self-heating [14].

Cosmetic masks are products for professional and home care, and recognized as a group of cosmetics designed for “extra skincare”. Among the marketed products, face masks in the form of emulsions, also referred to as cream face masks, have been enjoying considerable popularity with consumers. Masks of this type are distinctive for their very thick consistency. They contain hydrophobic and hydrophilic substances, rheology modifiers, emulsifiers, pH regulators, preservatives, fragrances, and dyes. After the application of face masks the skin should be well hydrated, glowing, smooth, soft, and fresh-looking. Commercially available face masks vary considerably in their form and composition, with both simple and complex formulations offered to consumers. Cosmetic companies are constantly on the lookout for new solutions to attract the attention of customers and satisfy their expectations. Also, there is a clear trend for an increasing use of ingredients of natural origin in products of this type.

While natural materials have been used for centuries, in recent years they have been receiving an unprecedented amount of interest. Companies are looking for innovative ingredients to differentiate themselves in the marketplace. One of the interesting ingredients is blue honeysuckle (*Lonicera caerulea var. edulis*), which has not yet been used on a larger scale in the cosmetic industry. This plant is known only in the food industry and is highly valued for its health-promoting effects [15,16,17,18,19,20]. On account of the rich composition blue honeysuckle exhibits, among others, antioxidant, moisturizing, and anti-inflammatory properties. It is a shrub producing high-quality berries that has been known for many centuries in China, Japan, and Russia. The native Ainu people of Hokkaido (Japan) called blue honeysuckle fruit the “elixir of life” because of its beneficial qualities. The plant comes in a number of varieties. According to the literature on the subject, it is also known by other common names such as sweetberry honeysuckle, blue fly honeysuckle, blue-berried honeysuckle, or honeyberry. Since blue honeysuckle is easy to cultivate, and its fruit offers a unique flavor and high nutritional value, it can be described without any exaggeration as the “berry of the future” [20,21,22]. Based on most recent studies, the plant reaches a height of 0.8 to 3 m, and can live up to about 25 years. Blue honeysuckle is not difficult to cultivate. It grows well on clay or sandy soils with a pH between 5 and 7. It is highly resistant to various diseases and pests, but also to low temperatures, being able to withstand frost down to −46 degrees Celsius [16]. The plant produces elongated fleshy fruit (berries), purple-black in color, covered with a bluish coating. The berries may be oval or cup-shaped, or cylindrical-ellipsoidal in shape. They contain very high levels of active substances, which is the reason why this rare shrub has been attracting such great interest. Blue honeysuckle fruit is rich in phenolic compounds, vitamins, and acids. Moreover, it is known to contain magnesium, calcium, potassium, and phosphorus [23]. Blue honeysuckle is also rich in polyphenols, i.e., secondary plant metabolites with highly diverse chemical structures. They contain aromatic rings linked by a heterocyclic system [21]. The compounds have very good antioxidant properties which contribute to slowing down the aging process. In addition, they are able to inhibit the oxidation of vitamin C, unsaturated fatty acids, and carotenoids. They also have antibacterial, anti-inflammatory, and chemoprotective effects. Based on the number of rings and how they are connected, they have been divided into flavonoids, phenolic acids, lignans, and stilbenes [15,20]. Flavonoids are very valuable antioxidants, and have two aromatic rings linked by a bond. A literature review shows that this group of polyphenols has beneficial effects on the human body and skin. Special attention should be paid to anthocyanins, which are present in large amounts in blue honeysuckle berries. Anthocyanins are a group of flavonoids that lower blood pressure, eliminate blood clots, and stimulate collagen and elastin synthesis. In addition, these compounds are known for their ability to increase the resistance of blood vessels and reduce their permeability, and produce an anti-inflammatory effect. Anthocyanins are primarily responsible for inhibiting lipid oxidation, which is a very important aspect from the viewpoint of cosmetology. Based on research, blue honeysuckle contains 244 mg/100 g of free catechins, 400 mg/100 g of procyanidins, 7.2 mg/100 g of rutin, 9.1 mg/100 g of isoquercetin, 2.8 mg/100 g of quercetin, and 11.6 mg/100 g of 7-glucoside luteolin [24]. In turn, phenolic acids found in the shrub have been shown to contain a carboxyl group and a hydroxyl group. They are divided into phenylacetic, cinnamic and benzoic acids. Phenolic acids also produce an antioxidant effect, which is a property linked to their structure. In addition, they protect body cells against damage. Acids (e.g., caffeic and ferulic) are able to block carcinogens formed during the metabolism of certain carcinogenic substances. According to Gazdik et al. [25] phenolic acids together with flavonoids largely determine the antioxidant properties of blue honeysuckle. In addition to polyphenols, blue honeysuckle contains large amounts of vitamin C, which has been used for many years in the cosmetic field, for example in skin-lightening products to control melanin production [26]. Vitamin C is known to have a host of health benefits. For example, it accelerates the production of hyaluronic acid and promotes the synthesis of collagen. It is worth emphasizing that ascorbic acid is an antioxidant, so it is often used in the formulation of cosmetics for mature skin. Based on its antioxidant properties, it helps protect the skin against photoaging and makes it firmer and more elastic. Of note, blue honeysuckle berries contain from 30.5 to 186.6 mg of ascorbic acid. A slightly different range is reported by Szot et al. [24], who assert that it varies from 48.4 to 66.1 mg/100 g. It is also worth highlighting that blue honeysuckle berries contain B vitamins: thiamine (vitamin B1), riboflavin (vitamin B2), pantothenic acid (vitamin B5), pyridoxine (vitamin B6), folic acid (vitamin B9), as well as nicotinamide (vitamin PP), biotin (vitamin H), and p-aminobenzoic acid (PABA) [16,20,21,22,23]. Vitamins of this group are increasingly used in the formulation of cosmetics because of their favorable impact on the condition of the skin. Special attention should be paid to nicotinamide, a potent antioxidant which reduces wrinkles, protects the skin barrier, and in addition improves the structure and color of the skin. Vitamin H, also found in blue honeysuckle, has anti-ageing properties, and slows down the production of sebum. Yet another ingredient, pantothenic acid, stimulates cell growth and regeneration, and accelerates the synthesis of proteins and lipids [20].

A number of authors, for example Kula et al. [27], highlight that blue honeysuckle can be a valuable source of health-promoting ingredients associated with a range of benefits, such as antimicrobial, anti-inflammatory, and antioxidant properties. As a result, they may provide protection against a range of diseases linked to bacterial infection, inflammatory processes or oxidative stress.

Blue honeysuckle is a source of multiple active substances, so in order for its valuable properties to remain intact, it must be incorporated into cosmetics in an appropriate form. From the cosmetic point of view, the optimal solution would be to add plant-derived raw materials into the formulation with as little processing as possible, which would allow complete preservation of valuable plant components.

This is one of the reasons why, in recent years, there has been a growing interest in mechanical comminution technologies resulting in products with desired particle sizes. The search is on for new methods of production to ensure not only an appropriate degree and uniformity of grain size in addition to and superior quality, but also to reduce the energy intensity of the manufacturing processes. These conditions are met by monochronic drying &powdering (“MDP”) [28,29,30,31], which is a technology developed by the company Admor Spółka Jawna (Poland).

MDP is an innovative method which has not yet been used on a global scale. It is a convenient technique for obtaining fully natural powders while maintaining the composition of the raw material together with all vitamins, polyphenols, and other bioactive substances. It needs to be stressed that powders obtained via this method are completely free of additives (preservatives, synthetic flavors, dyes or carriers). Raw materials for MDP processing are sourced from controlled plantations and undergo phytosanitary and microbiological inspections [28,29,30,31].

MDP involves drying raw materials at low temperatures (approximately 40 °C) with simultaneous micronization to ensure an appropriate level of particle size reduction and low water content. During the process of powder production, the capillaries in the hard parts of the raw material are opened mechanically for the duration of treatment, and water is evaporated simultaneously. The resulting processed raw material is resistant to high temperatures, which might cause deterioration of its quality. The mixture formed when water vapor combines with air creates a specific type of protective barrier, so that the dry portion of the raw material does not exceed 20–35 °C, or the upper limit of heat treatment. The temperature reached by the raw material treated by MDP is considerably lower than the temperature of heated air. Consequently, thermal energy introduced with the air into the treatment area is used for the evaporation of water present in the material. The water content is typically between 4 and 15%, and can be adjusted as desired. The process of raw material drying takes from about dozen minutes to an hour, and depends on the water content and expected final powder weight. Since the processing technique is very precise, whole fruitsand vegetables can be converted into powders while retaining their beneficial properties [28,29,30,31].

MDP is a technology that produces powders of superior quality to those obtained by the commonly used technique of freeze drying. Table 1 presents a comparison of both methods, listing their distinctive features.

The data listed in Table 1 demonstrate that MDP has a competitive advantage over freeze drying. Even though both technologies provide high quality powders without any additives, MDP is less expensive and does not require any special packaging. In addition, it ensures faster water removal and drying, and produces powders that are much more stable than those obtained through freeze drying.

Powders obtained by MDP are characterized by the following properties:

- 100% natural,
- no preservatives, dyes, carriers, or synthetic flavors,
- rich in bioactive compounds,
- low water content (6 to 8%),
- considerable size reduction (1 to 100 microns),
- stable in storage [28,29,30,31].

The method has applications not only in the cosmetic industry, but also in food production. MDP is suitable for processing vegetables, fruit, seeds, herbs, milk, meat, mushrooms, and even honey. The resulting powders are readily digestible and easily absorbed by the body. Being rich in various vitamins, fiber and polyphenols, they bring a range of health-enhancing benefits [28,29,30,31].

In the present study, blue honeysuckle powder obtained by an innovative method of low-temperature drying with simultaneous micronization was used in the formulation of skincare masks. There are no studies in the literature on the application of blue honeysuckle powder obtained by the innovative technique of MDP. However, considering that blue honeysuckle has a range of valuable properties, and this novel production method carries tangible benefits, the resulting powder might serve as a valuable ingredient in cosmetic masks.

The aims of the study were to develop face mask formulations with added blue honeysuckle powder obtained by applying an innovative method of drying combined with simultaneous micronization, and to determine the effect of the ingredient on the physicochemical and functional properties of products of this type. To this end, a range of tests were performed to determine parameters such as stability (temperature and centrifuge tests), dynamic viscosity, yield point, texture analysis, and evaluation of the color of cosmetics and degree of skin hydration.

## 2. Results and Discussion

### 2.1. Stability

Maintaining stability is a fundamental requirement for cosmetics formulated as emulsions. Cosmetic samples were visually evaluated prior to testing. No signs of instability were observed in any of them. Cosmetics were homogeneous. The base sample was characterized by a white color, and in the subsequent ones, with increasing concentration of blue honeysuckle, an increasingly clear pink color was observed (Table 2).

Stress tests confirmed the stability of all tested masks over time, both at low and high temperatures. Similarly, centrifuge tests revealed no signs of instability in cosmetic masks containing varying amounts of blue honeysuckle powder.

### 2.2. Dynamic Viscosity

Viscosity is one of the crucial aspects that are taken into consideration when evaluating the quality of face masks. It determines the functional properties of this type of cosmetics, e.g., spreadability on the skin of the face and neck, or easy dispensing from the package. In fact, high viscosity is frequently seen by consumers as a property indicating a high content of active ingredients in the formulation. However, the viscosity of cosmetics is determined to a large extent not by their active ingredients but rather by rheology modifiers and surfactants.

**Figure 1:**
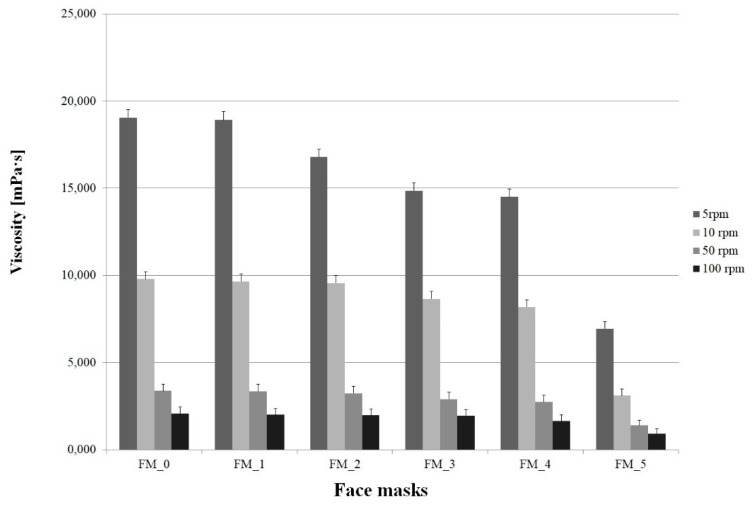
Dynamic viscosity of face masks with different concentrations of *Lonicera caerulea* Fruit Powder.

Measurements of dynamic viscosity of face masks with varying concentrations of blue honeysuckle powder were performed at different rotational speeds (5, 10, 50 and 100 rpm). The measurement results are shown in Figure 1.

The dynamic viscosity of the evaluated face masks at 5 rpm was in the range of 6940–19,060 mPa·s, at 10 rpm it was 3110–9790 mPa·s, at 50 rpm—1396–3365 mPa·s, and at 100 rpm—926–2066 mPa·s. The lowest value was recorded for the formulation P_5 which had the highest content of blue honeysuckle (5 rpm—6940 mPa·s, 10 rpm—3110 mPa·s, 50 rpm—1396 mPa·s, 100 rpm—926 mPa·s). The results warrant the conclusion that an increase in rotational speed is associated with a decrease in dynamic viscosity, and rising blue honeysuckle concentrations in the masks are linked to a drop in dynamic viscosity at all rotational speeds. These correlations are typical of the majority of cosmetic emulsions, and ensure appropriate spreadability on the skin [32].

The dynamic viscosity values of cosmetic masks reported in the literature vary within a very wide range, from 100 mPa·s even up to 350,000 mPa·s [3,13,33]. They depend on the physical form of the mask as well as its composition, including the concentration and type of rheology modifiers. For example, Hendrawati et al. [33] in their study evaluated the dynamic viscosity of face masks containing varying concentrations of Aloe Vera Gel. The measured values were in the region of 20,000 Pa·s. Similar values, approximately 25,000 mPa·s, were obtained by Klimaszewska et al. [13] in their study of skincare masks containing a blackberry extract obtained in supercritical carbon dioxide conditions. Higher values, i.e., approximately 140,000 and more, have been typically reported in the literature for peel-off masks [3].

### 2.3. Yield Point

The rheological characteristics of face masks largely determine their functional properties, significantly affecting the quality of this type of cosmetics. In addition to dynamic viscosity, a significant parameter is yield point. For the manufacturer, it is important for the selection of optimal packaging for the cosmetic and the dispensing method, which in turn contributes to the efficiency and ease of product use by the consumer. Lower values of the parameter indicate a lighter consistency and potentially better product spreadability on the skin.

**Figure 2:**
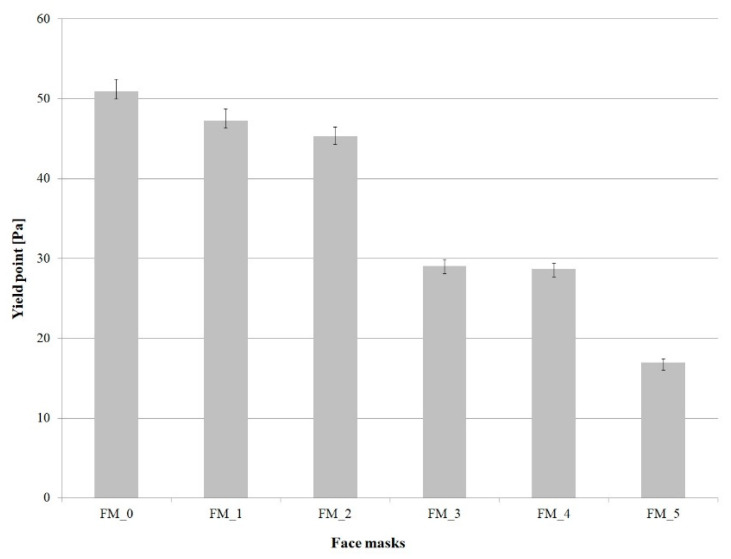
Yield point of face masks with different concentrations of *Lonicera caerulea* Fruit Powder.

Figure 2 shows the values of the yield point for face masks with varying concentrations of blue honeysuckle powder.

The test results indicate that the highest yield point (50.94 Pa) was recorded for the base mask (FM_0). Adding blue honeysuckle powder to skincare mask formulations led to a decrease in yield value compared with the reference sample. The lowest yield point (16.99 Pa) was observed for the formulation P_5, which hadthe highest content of blue honeysuckle powder (0.9%). It can be presumed that this formulation is characterized by the best skin spreadability.

Similarly to dynamic viscosity, the values of yield point reported for cosmetic masks in the literature vary within a wide range, from approximately 5 Pa to 400 Pa [13,34]. For example, Klimaszewska et al. [13] conducted a study to determine yield point values of cosmetic masks formulated as emulsions containing varying amounts of a blackberry extract. The values ranged from 30 to 70 Pa.

In contrast, high yield point values, approximately 400 Pa, are noted for face masks based on clays, e.g., Cameroonian mineral clays [34].

### 2.4. Texture Analysis

**Figure 3:**
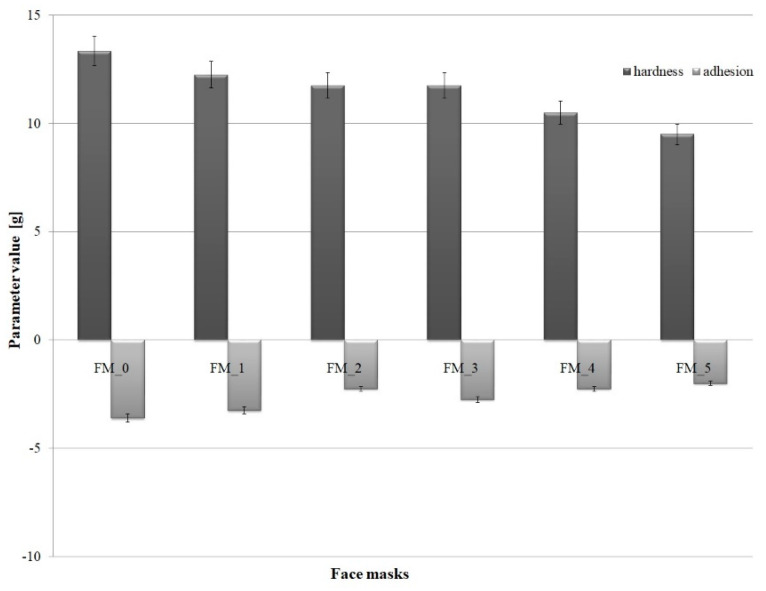
Hardness and adhesive force as a function of *Lonicera caerulea* Fruit Powder concentration in face masks.

Hardness and adhesive force, which characterize texture, are significant parameters from the viewpoint of application of cosmetic products. They enable easy spreading and product adherence to the skin, which is extremely important in such formulations as cosmetic masks. The chart below (Figure 3) shows the values of hardness and adhesive force recorded for different cosmetic formulations under study.

Figure 3 illustrates changes in the parameters describing texture (hardness and adhesive force) as a function of the concentration of blue honeysuckle powder in the formulation of face masks. The mask serving as a reference point (FM_0) had a hardness value of 13.34 g. After incorporating blue honeysuckle powder into the emulsions, the hardness level ranged from 12.25 g to 9.5 g. Thus, a reduction in the measured parameter was observed, by up to 30% relative to the standard, along with an increase in the proportion of the plant powder. From the viewpoint of application-related properties, the results are satisfactory. With respect to packaging, cosmetic face masks are usually offered in sachets, jars or squeeze tubes. Based on the measured data, it can be assumed that the products should be easy to dispense and apply to the skin.

The opposite trend was observed for adhesion. The value of adhesive force was found to decline along with increasing concentrations of Lonicera caerulea fruit powder. However, the recorded difference (−3.6 g to −2.0 g) is insignificant in the context of the type of measured parameter. The values obtained for adhesive force show that both the spreading and application of the cosmetic to the skin should present no problems for the user.

Klimaszewska et al. [13] reported a similar tendency. In their study, a gradual increase in the proportion of blackberry extract in the formulation of cosmetic masks led to a decrease in hardness and adhesive force. The hardness values obtained for the formulated masks varied between 4.5 g and 5.5 g, while adhesive force ranged from −1.5 to 1.0 g. Thus, they were several times lower than those recorded in the present analysis.

In another study [35], Klimaszewska et al. examined the impact of chamomile extract added at varying concentrations to an ointment formulation by evaluating the effect of the applied raw material on the texture of the samples, among other parameters. The hardness of the samples was found to have dropped from 179 g to 125 g after the incorporation of the plant material. A similar pattern was noted for adhesive force. The parameter decreased up to twofold as a function of increasing proportions of chamomile extract obtained in supercritical carbon dioxide conditions in an ointment formulation.

### 2.5. Determination of Color Parameters

Plant derivatives contain a wide range of active substances which may have the ability to change the color of emulsions (including cosmetic emulsions). Table 3 summarizes the values of parameters determined and calculated for the formulated cosmetic masks.

Adding blue honeysuckle powder to the formulated cosmetic mask emulsions was found to reduce the value of the measured parameter L (sample lightness) by up to 15% compared to the value obtained for the reference (L* _FM_0_).

For the parameter a*, the values changed from −1.03 (base formulation) to 6.48 (mask containing 0.9 wt.% of blue honeysuckle powder). In practical terms, this means that the intensity of the green color of the face masks decreases as the proportion of the plant material used increases, and the masks turn red.

The parameter b* was shown to increase from b* = 0.68 for the reference mask to b*_FM_5_ = 2.16 for the emulsion containing Lonicera caerulea fruit powder at c = 0.9 wt.%, so a slight increase in the intensity of the color yellow was noted.

Based on the determined L*, a* and b* values, differences in color saturation and variation in the hue of the formulated face masks were calculated (Table 3). Color saturation was shown to change from ΔC*_FM_1_= −0.08 in the emulsion containing 0.1 wt.% of blue honeysuckle to a value nearly 70 times higher for the sample formulated with 0.9 wt.% of Lonicera caerulea fruit powder. Of note, the greatest increase in the value of ΔC*_FM_ was seen after adding the powder at a concentration of 0.7 wt.%. Based on the data obtained, it can be concluded that an increase in the concentration of blue honeysuckle powder obtained by low-temperature drying in skincare face masks resulted in a brighter and more saturated color compared to the color of the reference sample.

With regard to differences in hue ΔH*, the tendency of change was the same as that observed for the values of ΔC*. Already, even the smallest dose of the plant powder (c = 0.1 wt.%) added to the cosmetic formulation brought about a difference in hue: ΔH*_FM_1_ = 1.92. Subsequent increases in the proportion of Lonicera caerulea fruit powder in the formulation produced further increases in the value of the measured parameter. However, it needs to be noted that the differences were no longer that significant, and the maximum recorded value of the parameter was ΔH*_FM_5_ = 5.22.

Looking at the numerical values listed in Table 3, a conclusion can be drawn that an increase in the concentration of Lonicera caerulea fruit powder in the formulated emulsions gave the samples a yellow-red color, with rising concentrations leading to a dominance of the red color and an increase in its saturation and hue.

There are known studies discussing the effect of blackberry seed extract added to face mask formulations on the color change of the cosmetic [13]. From the study results reported by Klimaszewska et al., it follows that an increase in the concentration of the extract in the formulated emulsions caused the samples to turn yellow-green, with the increase resulting in a dominance of the yellow color and an increase in its saturation.

In their study, Klimaszewska et al. [35] assessed the functional properties of an ointment as a function of the changing proportion of chamomile flower extract obtained under supercritical CO_2_ conditions. The study also demonstrated a significant change in color, saturation and hue after the incorporation of plant-derived additives. Adding the extract into the ointment was found to change the color of the samples to yellow-green. Green was the predominant color, and its saturation and hue varied between different ointment formulations.

Also, Bujak et al. [36] carried out colorimetric tests, which demonstrated that the addition of hydroalcoholic plant extracts to cosmetic emulsions determined changes in L*, a* and b* values as well as changes in color saturation and hue of the emulsions. Following the incorporation of extracts obtained from the leaves of globe amaranth (*Gomphrena globosa* L.), butterfly pea (*Clitoria ternatea* L.), safflower (*Carthamus tinctorius* L.), pomegranate (*Punica granatum* L.), and corn poppy (*Papaver rhoeas* L.), the color of the emulsion was found to turn from white to yellow, red, and blue-violet.

### 2.6. Skin Hydratation Level

Inadequate skin hydration may have serious health consequences. In people suffering from dry skin, even a minimum exposure to an irritant may lead to skin irritation. In most cases, affected individuals develop red rough skin or small vesicles filled with serous fluid [37,38,39,40]. Such cutaneous manifestations typically cause itching, burning, and in some cases also pain. Therefore, proper skin hydration is extremely important.

**Figure 4:**
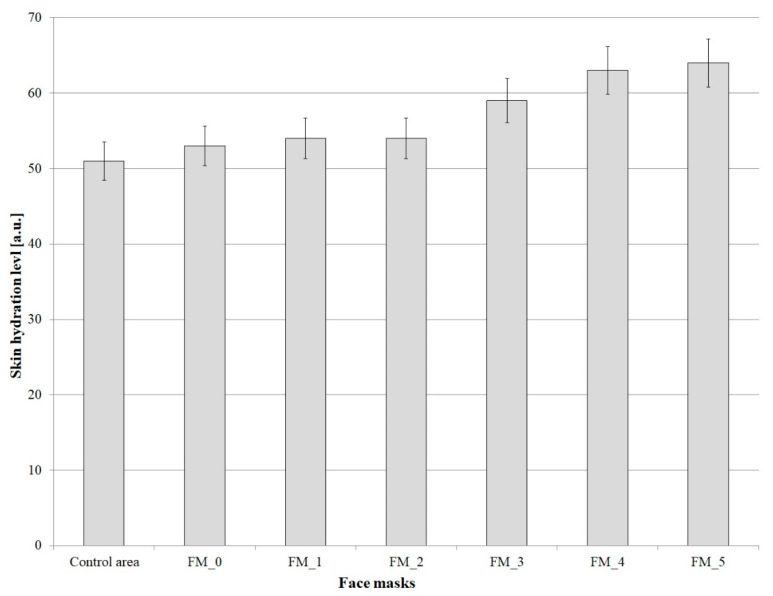
Skin hydration level after the application of face masks with varying concentrations of Lonicera caerulea fruit powder.

Figure 4 shows the results of skin hydration tests after the application of face masks with varying concentrations of blue honeysuckle powder.

The skin hydration level in the control area was approximately 51 a.u. The application of the base mask resulted in a 3.9% increase in hydration compared to the control area. Following the addition of 0.1% of blue honeysuckle powder, the level of skin hydration was found to have improved by 6.1% compared to the control area. Another increase in the concentration of the analyzed raw material (to 0.3%) led to an improvement in skin hydration by 6.7%, while adding blue honeysuckle powder at concentrations of 0.5%, 0.7% and 0.9% was shown to increase hydration relative to the control point by 17.1%, 25.9%, and 27.3%, respectively. The highest increase in skin hydration (by 13.93 a.u.) was noted after the application of the formulation FM_5. A comparison of the formulations containing blue honeysuckle powder with the control point and the base mask formulation reveals that the material improves the level of skin hydration. Based on the test results, it can be concluded that an increase in the content of blue honeysuckle in cosmetic masks translates into an increase in the degree of skin hydration. Following the application of cosmetic masks to the skin, an improvement in skin moisture is attributed to the properties of blue honeysuckle as well as the occlusive effect. The skin hydration levels determined after using the face masks show that the skin is properly moisturized. This is an extremely important aspect, as face masks are expected to be more effective in terms of their skin hydrating properties than, for example, face creams [41].

## 3. Materials and Methods

### 3.1. Materials

**Figure 5:**
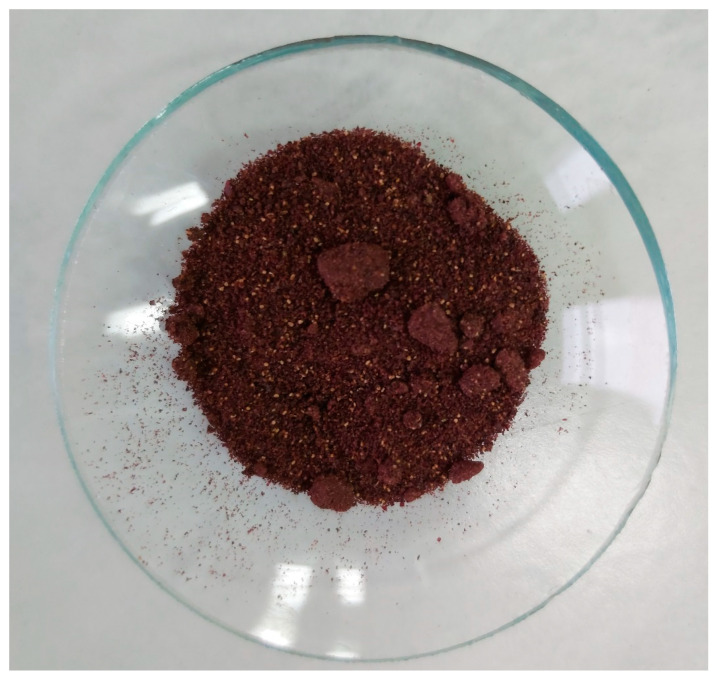
Picture of blue honeysuckle powder.

As ingredients of masks, the following materials were used:

- Cetearyl Alcohol, Lanette O from BASF Poland;
- Sweet Almond Oil from PPH Standard Poland;
- Cera Alba from PPH Standard Poland;
- Caprylic/Capric Triglicerides, Crodamol GTCC from Croda Poland;
- Urea from PPH Standard Poland;
- Sodium Benzoate and Potassium Sorbate, KEM BS from Pol Nil S.A.;
- Lactic Acidfrom HSH Chemie Poland;
- Polyglyceryl-4 Laurate, Natragem E145 NP from Croda Poland;
- Lonicera Caerulea Fruit Powder from Admor Poland (Figure 5).

### 3.2. Cosmetic Mask Formulations

**Figure 6:**
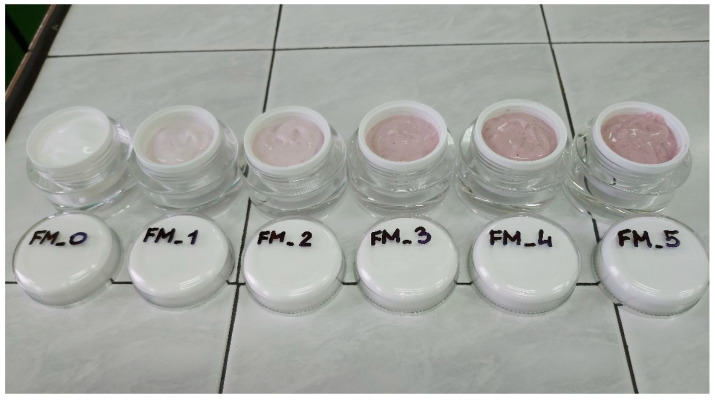
Cosmetic masks containing varying concentrations of blue honeysuckle powder.

Cosmetic mask formulations were developed on the basis of the relevant literature and the author’s ownexperience in the field [2,3,4,5,6,7,8,9,10,11,12,13,14]. A total of six formulations were prepared (Figure 6), containing varying concentrations of blue honeysuckle (INCI: *Lonicera Caerulea Fruit Powder*). The face mask formulations are presented in Table 4.

### 3.3. Formulation Procedure

Phase I ingredients were measured out and combined, and then stirred until complete dissolution on a magnetic stirrer while being heated in a water bath to a temperature of approximately 70–80 °C. Phase II ingredients were weighed into another beaker, and stirred until complete dissolution on a magnetic stirrer while being heated in a water bath to a temperature of approximately 70–80 °C. Once both phases reached the same temperature, they were combined, with phase I added to phase II. The mixture was stirred for a while on a magnetic stirrer in a water bath, then stirred and simultaneously cooled down to approximately 30 °C, and homogenized (Silent Crusher M homogenizer from Heidoplph) at a temperature of approximately 30 °C at a speed of 10 rpm for 5 min. Next, a preservative was added and the pH was adjusted to 5.5. In the final step, blue honeysuckle was incorporated into the formulation, and the whole mixture was stirred for 3 min on a magnetic stirrer.

### 3.4. Methods

#### 3.4.1. Stability

To investigate the stability of the formulated skincare face masks, temperature stress tests were conducted in order to visually assess the stability of the cosmetics stored alternately at elevated (40 °C, 1 day) and reduced (5 °C, 1 day) temperatures. The tests were carried out in a ST-68 type incubator and an Amica refrigerator. The test took eight days (four full cycles) to complete. In addition, the cosmetic samples were evaluated for their resistance to centrifugal force using a Rotofix 32 A centrifuge from Zentrifugen. The test took 30 min and was carried out at 2000 rpm [13,42,43].

#### 3.4.2. Dynamic Viscosity

The dynamic viscosity of the masks was measured using a Brookfield HA DV III Ultra viscometer. The test was conducted at approximately 20 °C, using Helipath spindles, with rotating spindle speeds of 5, 10, 50, and 100 rpm.

#### 3.4.3. Yield Point

The yield point of the face emulsions was evaluated using a Brookfield HA DV III Ultra viscometer. The instrument was equipped with a set of vane spindles. A constant spindle speed (5 rpm) was selected at which the measurements were performed. The minimum value of shear stress above which the masks under study became more fluid corresponded to the yield point. Data were recorded and analyzed using EZ-Yield Software.

#### 3.4.4. Texture Analysis

Texture was evaluated using a Brookfield CT3 texture analyzer. A spherical nylon probe, 25.4 mm in diameter, was used. The probe penetration depth was 10 mm at a constant head movement speed of 0.1 mm/s. The results were recorded with Texture Pro CT software. Texture profile analysis (TPA) assessed a range of properties including hardness, i.e.,the mass required to press the probe to a depth of 10 mm (maximum force recorded during a single test cycle), and adhesive force, i.e., the measure of adhesion of the formulation to the probe (mass that needs to be applied to the probe to remove it).

#### 3.4.5. Determination of the Color Parameters

Color tests were performed using a CR 400 colorimeter from Konica Minolta. Each formulation was assessed in the C.I.E. system based on the measurement of three trichromatic components: L, a* and b*. Each color was determined through three components:

L*—lightness (intensity of color brightness; by comparing their L* values, colors can be classified as either light or dark),
a*—value between red and green,
b*—value between yellow and blue.

Next, on the basis of obtained data, differences in color saturation (ΔC__FM_) and hue (ΔH__FM_) were calculated from the formula below, where:

$∆C_{FM\_0\;–FM\_X}^{*}$—difference in color saturation between the base mask formulation and the face mask containing blue honeysuckle,
FM___0—base mask formulation,
FM___X—face mask with blue honeysuckle at a concentration of X,
X = 0.1; 0.3; 0.5; 0.7; 0.9.

The color saturation of the base mask and the masks containing blue honeysuckle was calculated from the Formulas (1) and (2):

(1) $$C_{FM_{0}}^{*}=\sqrt{{\left(a_{FM_{0}}^{*}\right)}^{2}+{\left(b_{FM_{0}}^{*}\right)}^{2}}$$

(2) $$C_{FM\_X}^{*}=\sqrt{{\left(a_{FM\_X}^{*}\right)}^{2}+{\left(b_{FM\_X}^{*}\right)}^{2}}$$

Next, the difference in hue was determined between the base mask and the formulation containing blue honeysuckle $∆H_{FM\;0\;–FM\;X}^{*}$ calculated from the Formula (3) below, where:

(3) $$∆H_{FM\_0\;–FM\_X}^{*}=\sqrt{{\left(∆E_{FM\_0–FM\_X}^{*}\right)}^{2}-{\left(∆L_{FM\_0-FM\_X}^{*}\right)}^{2}-{\left(∆C_{FM\_0–FM\_X}^{*}\right)}^{2}}$$

where: $∆E_{FM\;0\;–FM\;X}^{*}$—difference in color between the base mask formulation (5) and the mask containing blue honeysuckle (6), calculated from the Formula (4):

(4) $$∆E_{FM\_0\;–FM\_X}^{*}=E_{FM\_0}^{*}-E_{FM\_X}^{*}$$

(5) $$E_{FM\_0\;}^{*}=\sqrt{{\left(L_{FM\_0}^{*}\right)}^{2}+{\left(a_{FM\_0}^{*}\right)}^{2\;}+{\left(b_{FM\_0}^{*}\right)}^{2}}$$

(6) $$E_{FM\_X}^{*}=\sqrt{{\left(L_{FM\_X}^{*}\right)}^{2}+{\left(a_{FM\_X}^{*}\right)}^{2}+{\left(b_{FM\_X}^{*}\right)}^{2}}$$

$∆L_{FM\;0\;–FM\;X}^{*}$—difference in lightness between the base mask formulation and the mask containing blue honeysuckle, calculated from the Formula (7):

(7) $$∆L_{FM\_0\;–FM\_X}^{*}=L_{FM\_0\;\;}^{*}-L_{FM\_X}^{*}$$

#### 3.4.6. Skin Hydration Level

The level of skin hydration was evaluated using a Corneometer CM 825, an instrument which determines stratum corneum capacitance. The measurement was performed on clean, degreased skin, which served as a point of reference. In the next step, 1 g of the skincare mask was applied to the skin on the forearm (an area measuring 20 × 20 mm), and left for approximately 10 min. After that time, the remains of the mask were removed with a cotton pad. Following 2 h at room temperature (22 °C), the level of skin hydration was measured. The test was conducted in a group of 50 women aged 35 to 40 years.

#### 3.4.7. Statistical Analysis

The bars in the graphs represent the arithmetic means of the values from three independent measurements. Confidence intervals representing the measurement error for a confidence level of 0.90 were determined. The error values are shown in the graphs.

## 4. Conclusions

The aims of the study were to develop skincare face mask formulations containing blue honeysuckle in the form of powder obtained by an innovative method of low-temperature drying with simultaneous micronization, and to determine the effect of the concentration of this ingredient on the physicochemical and functional properties in cosmetic products of this type.

To this end, a range of tests were carried out to assess stability (temperature and centrifugal tests), dynamic viscosity, yield point, texture analysis, color assessment of cosmetics, and the degree of skin hydration before and after the application of the formulated cosmetics.

Based on the results of laboratory tests, the following conclusions can be drawn:

- All test formulations were stable over time, so the addition of blue honeysuckle does not adversely affect the stability of cosmetic face masks.
- Dynamic viscosity of the test formulations was in the range of 920 to 19,000 mPa·s. An increase in the concentration of blue honeysuckle was found to cause a decrease in the value of dynamic viscosity at all speeds, resulting in the development of a product with a lighter consistency. It was also noted that an increase in rotational speed was linked to a decrease in dynamic viscosity for all face masks tested.
- An increase in the concentration of blue honeysuckle powder produces a decrease in yield point. Consequently, the face masks are easier to spread on the skin.
- The hardness of the formulations under study decreased along with increasing concentrations of blue honeysuckle in the face masks, and ranged from 9.5 to 13.34 g. Similarly to hardness, changes in adhesive force were also observed, with values varying within a range of −2 to −3.6 g. The results were found to correlate with those obtained in the dynamic viscosity and yield point tests.
- The highest degree of whiteness was determined in the base formulation, but as the content of blue honeysuckle in the cosmetic masks increased, the parameter L was seen to decrease. With regard to the change in the chromatic color components “a” and “b”, it can be concluded that an increase in the concentration of Lonicera caerulea fruit powder in the formulated emulsions caused the masks to turn reddish-yellow, while an increase in the concentration of plant powder in the formulation resulted in a dominance of the red color, inducing a change in saturation and hue.
- The application of skincare masks containing blue honeysuckle improves the level of skin hydration. An analysis found that the highest degree of improvement in this parameter was achieved after the application of the face masks containing 0.7% and 0.9% of the raw material under study. Consequently, the application of such formulations, enriched with blue honeysuckle, may contribute to an overall enhancement of skin condition. Crucially, an increase in the content of blue honeysuckle in cosmetic masks translates into an improvement in skin hydration.

In sum, it can be concluded that the aim of the study was achieved. Skincare face masks formulated with the addition of blue honeysuckle powder may provide inspiration for cosmetic companies. The valuable properties of this innovative raw material offer great opportunities to the cosmetic industry.

Since blue honeysuckle powder used in face masks has beneficial effects on studied physicochemical and functional properties, it will be the subject of further research. The authors plan to extend the research by performing tests of physical and chemical characteristics and determining the composition of active substances contained in Lonicera caerulea fruit powder obtained by an innovative method of drying with simultaneous micronization. In addition, the PAO mark for this type of cosmetics will be made, and the microbiological stability (challenge test) and efficacy tests (antimicrobial and anti-inflammatory) of the developed masks will also be performed.

## Figures and Tables

**Table 1:** Comparison of freeze drying and MDP [28,29,30,31].

Feature	Freezedrying	MDP Technology
Price	High	Low
Packaging	Special packaging	No specific packaging requirements
Waterremoval	Initial freezing followed by ice cube removal by conversion into water vapor	By evaporation with simultaneous comminution
Dryingtime	Additional drying between 3 and 6 h	About dozen minutes up to an hour
Incorporation of additives (includingcarriers)	None	None
Stability of storage of resultingpowders	Unstable	Stable
Comminution	Before or after freeze drying	Simultaneously

**Table 2:** Results of visual evaluation of cosmetic masks containing different concentrations of blue honeysuckle powder after stress and centrifuge tests.

Formulation	Parameter			
	Stability	Homogeneity	Color	Odor
**FM_0**	Stable	Homogeneous	White	Neutral, noticeable oily odor
**FM_1**		Visible particles of blue honeysuckle	Light pink	Noticeable delicate smell of blue honeysuckle
**FM_2**				
**FM_3**			Pink	
**FM_4**				
**FM_5**				

**Table 3:** Results of colorimetric measurements of cosmetic masks with varying concentrations of *Lonicera Caerulea* Fruit Powder.

Formulation	Parameter				
	L*	a*	b*	Difference in Color Saturation (ΔC*_FM_)	Difference in Hue (ΔH*_FM_)
FM_0	91.87	−1.03	0.68	-	-
FM_1	88.72	0.90	0.72	−0.08	1.92
FM_2	82.15	2.60	0.93	1.52	3.30
FM_3	81.88	3.92	1.65	3.01	4.04
FM_4	79.00	5.81	2.00	4.91	4.94
FM_5	78.20	6.48	2.16	5.59	5.22

**Table 4:** Formulations of cosmetic face masks with varying concentrations of blue honeysuckle powder.

Phase	Ingredients [INCI]	Symbol of Mask					
		FM_0	FM_1	FM_2	FM_3	FM_4	FM_5
		Concentration [% *w*/*w*]					
**I**	Polyglyceryl-4 Laurate	1.4					
	Cetearyl Alcohol	6.0					
	Glyceryl Stearate	5.0					
	Sweet Almond Oil	2.0					
	Cera Alba	3.0					
	Caprylic/Capric Triglicerides	5.0					
**II**	Aqua	at 100					
	Urea	1.0					
**III**	Sodium Benzoate and Potassium Sorbate	1.0					
	Lactic Acid	to pH = 5.5					
	Lonicera Caerulea Fruit Powder	-	0.1	0.3	0.5	0.7	0.9

## Data Availability

Data is contained within the article.

## Sample Availability

Samples of the compounds used and the plants extracts tested are availablefrom the authors.
